# SIRT5 deficiency exacerbates sepsis acute renal injury by activating ACSL4/FTH1 axis dependent ferroptosis

**DOI:** 10.1371/journal.pone.0361332

**Published:** 2026-10-27

**Authors:** Liliang Jiang, Xi Yu, Guang Shan, Ziqi He, Yongbo Liu, Wenlong Lin, Lingling Song, Zhongyuan Xia

**Affiliations:** 1 Department of Anesthesiology, Renmin Hospital of Wuhan University, Wuhan, Hubei, China; 2 Department of Urology, Renmin Hospital of Wuhan University, Wuhan, China; University of Waterloo, CANADA

## Abstract

Sepsis‑associated acute kidney injury (S‑AKI) is a major cause of morbidity and mortality in critically ill patients, yet effective targeted therapies remain lacking due to an incomplete understanding of its pathogenesis. Ferroptosis, an iron‑dependent form of regulated cell death driven by lipid peroxidation, has recently been implicated in AKI, but the upstream regulators of this process in sepsis remain undefined. Here, we identify SIRT5, a mitochondrial NAD ⁺ ‑dependent desuccinylase, as a critical endogenous suppressor of ferroptosis in S‑AKI. Using a cecal ligation and puncture (CLP) murine model and lipopolysaccharide (LPS)‑stimulated HK‑2 proximal tubular cells, we show that SIRT5 expression is significantly downregulated in septic kidney injury. Renal tubular‑specific ablation of Sirt5 exacerbated CLP‑induced renal dysfunction, structural damage, and tubular cell death, whereas AAV9‑mediated SIRT5 overexpression attenuated these pathological changes. Mechanistically, SIRT5 deficiency tipped the balance of the ACSL4/FTH1 axis toward ferroptosis: it increased ACSL4‑dependent membrane lipid remodeling and reduced FTH1‑mediated iron sequestration, accompanied by suppression of the antioxidant enzyme GPX4. Consequently, lipid peroxidation, iron overload, and mitochondrial damage were markedly amplified. Notably, knockdown of ACSL4 in SIRT5‑deficient HK‑2 cells effectively reversed lipid peroxidation, iron accumulation, and cell death, establishing ACSL4 as a key downstream effector of SIRT5‑mediated ferroptosis suppression. Our findings reveal a previously unrecognized SIRT5–ACSL4/FTH1 regulatory axis that governs ferroptotic susceptibility in renal tubular cells during sepsis, and suggest that pharmacological restoration of SIRT5 activity or blockade of ACSL4‑driven ferroptosis may offer novel therapeutic avenues for S‑AKI.

## Introduction

Sepsis is a life-threatening condition characterized by a dysregulated host response to infection and subsequent organ dysfunction. Among its complications, sepsis-associated acute kidney injury (S-AKI) is one of the most frequent and clinically severe forms of organ injury, accounting for a substantial proportion of acute kidney injury cases in critically ill patients [1–4]. S-AKI is associated with prolonged intensive care unit stay, increased in-hospital mortality, and a higher risk of chronic kidney disease [5,6]. Despite advances in supportive care, there remains a lack of effective targeted therapies for S-AKI, largely because its molecular pathogenesis is not fully understood.

Sirtuin 5 (SIRT5) is a mitochondrial NAD ⁺ -dependent enzyme that regulates protein succinylation, malonylation, and glutarylation. As an important modulator of mitochondrial metabolism and stress adaptation, SIRT5 has been shown to exert protective effects in several disease models, including ischemic injury, metabolic disorders, and renal damage. Previous studies have suggested that SIRT5 deficiency may worsen oxidative stress and inflammatory injury in the kidney [7–9]. However, its specific role in S-AKI has not been fully characterized.

Ferroptosis is a regulated form of cell death driven by iron-dependent lipid peroxidation and oxidative membrane damage [10,11]. Increasing evidence indicates that ferroptosis contributes to the pathogenesis of AKI, including septic kidney injury. The susceptibility of cells to ferroptosis is tightly controlled by iron metabolism, lipid remodeling, and antioxidant defense systems. Ferritin heavy chain 1 (FTH1) is a major intracellular iron-storage protein that limits the labile iron pool and protects cells from iron-mediated oxidative injury [12]. In contrast, acyl-CoA synthetase long-chain family member 4 (ACSL4) promotes ferroptosis by enriching membrane phospholipids with polyunsaturated fatty acids, thereby increasing the substrate available for peroxidation [13,14]. Recent studies have demonstrated that ferroptosis in renal tubular epithelial cells is a key driver of S-AKI progression [15,16]. Preliminary evidence suggests that sirtuins may modulate ferroptotic pathways by regulating the stability or activity of key ferroptosis-related proteins [17,18]. Although emerging evidence indicates that sirtuins may regulate ferroptosis, whether SIRT5 modulates ferroptotic signaling in S-AKI remains unknown.

In the present study, we hypothesized that SIRT5 protects against S-AKI by suppressing ferroptosis through regulation of the ACSL4/FTH1 axis. Using CLP-induced septic mice, renal tubular-specific Sirt5 knockout mice, AAV9-mediated SIRT5 overexpression, and LPS-treated HK-2 cells, we investigated the role of SIRT5 in septic kidney injury and explored the underlying mechanism. Our findings reveal that SIRT5 deficiency aggravates S-AKI by promoting ACSL4-mediated lipid peroxidation and impairing FTH1-dependent iron storage, thereby identifying the SIRT5/ACSL4/FTH1 axis as a potential therapeutic target in septic kidney injury.

## Materials and methods

### Animals and treatment

Male C57BL/6 mice (aged 8–10 weeks, weighing 20–25 g) were obtained from Beijing HFK Bioscience Co., Ltd. All animals were housed under specific pathogen‑free conditions with a 12‑h light/dark cycle and ad libitum access to food and water. All animal protocols, including the renal tubule-specific SIRT5 knockout model (SIRT5^(flox/flox); Cdh16-Cre) and the AAV9-mediated SIRT5 overexpression model, were approved by the Ethics Committee of Renmin Hospital of Wuhan University (approval no. 20250205A). At the experimental endpoint, mice were humanely euthanized by gradual carbon dioxide inhalation in a sealed chamber, followed by confirmation of respiratory arrest and death.

### Renal tubule‑specific SIRT5 knockout mice

To achieve conditional deletion of SIRT5 specifically in renal tubular epithelial cells, we employed the Cre‑loxP recombination system. SIRT5‑floxed mice (SIRT5^(flox/flox)) and Cdh16‑Cre transgenic mice were obtained from Saiye Biotechnology Co. (Guangzhou, China). In Cdh16‑Cre mice, Cre recombinase expression is driven by the mouse cadherin 16 (Cdh16) promoter, which directs Cre activity selectively to renal tubular epithelial cells in adult kidneys. By crossing SIRT5^(flox/flox) mice with Cdh16‑Cre mice, we generated offspring harboring homozygous floxed SIRT5 alleles together with the Cdh16‑Cre transgene (SIRT5^(flox/flox); Cdh16‑Cre), resulting in renal tubule‑specific SIRT5 deficiency. Genotyping was performed by PCR using tail genomic DNA, and knockout efficiency was verified by Western blot and qRT‑PCR analysis of kidney tissues. SIRT5^(flox/flox) littermates without the Cdh16‑Cre transgene were used as wild‑type controls.

### AAV9‑mediated SIRT5 overexpression in vivo

To manipulate SIRT5 expression in vivo, adeno‑associated virus serotype 9 (AAV9) vectors were utilized. The coding sequence of mouse SIRT5 was cloned into the AAV9 vector to generate rAAV9‑SIRT5. A control empty vector (rAAV9) was used as a negative control. A total of 100 μL of virus (1 × 10^12^ viral genome particles/mL) was injected via the tail vein 3 weeks prior to CLP surgery. Successful transduction and SIRT5 overexpression in kidney tissues were verified by Western blot and qRT‑PCR analysis at the experimental endpoint.

### Sepsis‑associated acute kidney injury model (CLP)

S‑AKI was induced in mice by cecal ligation and puncture (CLP) as previously described. Briefly, mice were anesthetized with isoflurane inhalation, and a midline abdominal incision was made. The cecum was exteriorized, ligated at approximately 50% of its length with a 4‑0 silk suture, and punctured twice with a 22‑gauge needle. The cecum was then gently returned to the abdominal cavity, and the incision was closed in layers. All mice received 1 mL of pre‑warmed normal saline subcutaneously for fluid resuscitation immediately after surgery. Sham‑operated mice underwent the same procedure except that the cecum was neither ligated nor punctured. Mice were monitored postoperatively and euthanized at 24 h after surgery for tissue and blood collection.

### Cell culture and treatment

Human proximal tubular epithelial cells (HK-2) and HEK293T cells were obtained from the American Type Culture Collection (ATCC). Cells were cultured in DMEM/F12 medium supplemented with 10% fetal bovine serum (FBS) and 1% penicillin/streptomycin at 37°C in a 5% CO_2_ atmosphere. To mimic sepsis in vitro, HK-2 cells were treated with LPS (10 μg/mL) for 24 hours.

For gene knockdown in vitro, cells were transfected with short hairpin RNA (shRNA) targeting SIRT5 (sh-SIRT5), ACSL4 (sh-ACSL4) using Lipofectamine 3000 (Invitrogen) according to the manufacturer’s instructions. To inhibit ferroptosis pharmacologically, cells were treated with Ferrostatin-1 (Fer-1, 1 μM) 2 hours prior to LPS stimulation.

### Renal histopathology and immunofluorescence

Kidney tissues were fixed in 4% paraformaldehyde, embedded in paraffin, and sectioned at 4‑μm thickness. Sections were stained with hematoxylin and eosin (H&E) to assess tubular injury. For histological scoring, at least five randomly selected high‑power fields (×400 magnification) per kidney section were examined by two independent investigators blinded to the experimental groups. Tubular injury was evaluated based on the following four parameters: (1) tubular dilation, (2) brush border loss, (3) cast formation, and (4) tubular necrosis. Each parameter was scored on a scale of 0–3 as follows: 0, no injury; 1, < 25% of tubules affected; 2, 25–50% of tubules affected; 3, > 50% of tubules affected. The total tubular injury score for each field was calculated as the sum of the four individual parameter scores (range 0–12), and the average score from the two investigators was used for statistical analysis.

For immunofluorescence (IF), sections were deparaffinized, rehydrated, and subjected to antigen retrieval. Sections were blocked with 5% BSA and incubated overnight with primary antibodies against SIRT5, ACSL4, or AQP1 (a proximal tubule marker). After washing, sections were incubated with fluorophore-conjugated secondary antibodies and DAPI. Images were captured using a laser scanning confocal microscope (Leica).

### Transmission electron microscopy (TEM)

To evaluate mitochondrial morphology characteristic of ferroptosis, small pieces of kidney cortex were fixed in 2.5% glutaraldehyde at 4°C overnight. Samples were post-fixed with 1% osmium tetroxide, dehydrated in graded ethanol, and embedded in epoxy resin. Ultrathin sections were stained with interactions and lead citrate and observed under a transmission electron microscope (Hitachi) to identify signs of ferroptosis, such as shrunken mitochondria, increased membrane density, and reduction of cristae.

### Biochemical assays

BUN levels were assessed by a BUN assay kit (Nanjing Jiancheng Bioengineering Institute). Serum samples were diluted as necessary and mixed with the provided reagents before incubation at room temperature. A microplate reader was then used to measure absorbance at 570 nm. sCr levels were determined using a commercial assay kit (Nanjing Jiancheng Bioengineering Institute) by incubating serum samples with the assay reagents and monitoring the reaction at 510 nm.

To assess oxidative stress and ferroptosis, the levels of Malondialdehyde (MDA), Glutathione (GSH) were measured using the MDA assay kit (JL-T0761) and the GSH

assay kit (JL-T0906) strictly following the manufacturer’s instructions (Jonlnbio, Shanghai, China). ferrous iron (Fe^2+^) (E-BC-K772-M) were used (Elabscience, Wuhan, China) in kidney tissues or cell lysates were determined using specific assay kits (Abcam) according to the instructions.

### ROS measurement

Intracellular reactive oxygen species (ROS) levels were assessed using the fluorescent probe DCFH-DA (C10444, Invitrogen, USA). After washing twice with phosphate-buffered saline (PBS), cells were loaded with 10 μM DCFH-DA and incubated at 37 °C for 20 minutes in the dark. Subsequently, ROS-associated fluorescence was acquired either by flow cytometry (Beckman Coulter, Brea, CA, USA) or by inverted fluorescence microscopy (Olympus IX83, Tokyo, Japan). Relative fluorescence intensity of ROS was quantified with FlowJo v10.6.2 (FlowJo LLC, Ashland, OR, USA) and ImageJ v1.53t (National Institutes of Health, Bethesda, MD, USA), respectively.

### Cell viability assay

HK-2 cells were cultured in 96-well plates (100 μL per well) at 37 °C with 5% CO_2_ until the density reached 70%, and Fer-1 (HY-100579, MCE, USA) was treated before harvest. Following a 12-hour incubation period, 100 μl of the CCK8 working solution (BS350B, Biosharp, China) was added to each well, and the mixture was then incubated for two hours at 37 °C. A microplate reader (EnSight, PerkinElmer, USA) was used to measure absorbance at 450 nm.

### Western blot analysis

Total protein was extracted from kidney tissues or HK-2 cells using RIPA lysis buffer containing protease and phosphatase inhibitors (Beyotime). Protein concentration was determined using a BCA Protein Assay Kit. Equal amounts of protein (30 μg) were separated by 10% SDS-PAGE and transferred onto PVDF membranes. The membranes were blocked with 5% non-fat milk and incubated overnight at 4°C with primary antibodies against SIRT5 (1:1000), ACSL4 (1:1000), FTH1 (1:1000), GPX4 (1:1000), GAPDH (1:1000), and β-actin (1:5000). After washing, membranes were incubated with HRP-conjugated secondary antibodies for 1 hour at room temperature. Protein bands were visualized using an ECL chemiluminescence kit and quantified using ImageJ software.

### Total RNA extraction and reverse transcription-quantitative PCR (RT-qPCR)

RNAiso Plus reagent (cat. no. 9108Q; Takara Bio, Inc.) was used for RT-qPCR analysis. cDNA was synthesized from RNA using Invitrogen RT kit (cat. no. K1621; Thermo Fisher Scientific, Inc.) and stored. RT-qPCR was conducted with a kit (cat. no. E096-01A; Novoprotein Scientific, Inc.), using GAPDH as an internal control for normalization. The PCR protocol included an initial 95˚C pre-denaturation for 30 sec, followed by 40 cycles of 95˚C denaturation for 5 sec and 60˚C annealing/extension for 30 sec, concluding with melt curve analysis to verify amplification specificity. Relative gene expression levels were calculated using the comparative 2^-ΔΔCq^ method. The specific primers used were as follows: SIRT5 forward 5’-AACGCAAAGCACATAGTCAT-3’ and reverse 5’-AAGCAAAGGCCAGAGGAGT-3’; and GAPDH forward 5’-GTCTCCTCTGACTTCAACAGCG-3’ and reverse 5’-CAGAGGAGACTGAAGTTGTCGC-3’.

### Fluorescence TUNEL assay

Apoptotic cells in kidney tissue sections were detected by fluorescence-based terminal deoxynucleotidyl transferase dUTP nick end labeling (TUNEL) staining using a commercial detection kit (Cat. #HY-K1078, MedChemExpress LLC, China) according to the manufacturer’s protocol. Briefly, paraffin-embedded kidney sections were deparaffinized in xylene and rehydrated through a graded ethanol series (100%, 95%, 85%, 70%, v/v) to distilled water. The slides were then incubated with proteinase K working solution (20 μg/mL in 10 mM Tris-HCl, pH 7.4–8.0) at 37°C for 15–20 min for antigen retrieval and permeabilization. After washing twice with phosphate-buffered saline (PBS), the sections were equilibrated with equilibration buffer at room temperature for 10–30 min. The TUNEL reaction mixture was freshly prepared by mixing the nucleotide mix, recombinant terminal deoxynucleotidyl transferase (rTdT) enzyme, and incorporation buffer containing fluorescein-labeled dUTP in appropriate proportions. Each tissue section was covered with 50 μL of the TUNEL reaction mixture and incubated in a humidified dark chamber at 37°C for 60 min. The reaction was terminated by washing the slides three times with PBS. Nuclei were counterstained with DAPI at room temperature for 10 min in the dark. After a final PBS wash, the sections were mounted with antifade mounting medium and sealed with coverslips. Fluorescence images were acquired using a fluorescence microscope (Olympus IX83, Tokyo, Japan). TUNEL-positive cells were identified by green fluorescence localized to the nuclei and quantified in five randomly selected high-power fields (×200 or ×400 magnification) per section. The apoptotic index was expressed as the percentage of TUNEL-positive cells relative to the total number of DAPI-stained nuclei. All analyses were performed by an investigator blinded to the experimental groups.

### Protein extraction and trypsin digestion

Renal cortical tissues were collected from mice in the sham group (n = 5) and the CLP group (n = 5) at 24 hours after surgery. Samples were rapidly frozen in liquid nitrogen and ground into a fine powder, which was then transferred into a 5-mL centrifuge tube. Four volumes of lysis buffer (8 M urea supplemented with 1% protease inhibitor cocktail, v/v) were added, and the mixture was sonicated on ice for 3 min using a high-intensity ultrasonic processor (Scientz). The lysate was centrifuged at 12,000 g for 10 min at 4°C to remove insoluble debris, and the supernatant was collected. Protein concentration was determined with a BCA assay kit following the manufacturer’s protocol. For protein precipitation, trichloroacetic acid was slowly added to the supernatant to a final concentration of 20% (w/v), vortexed thoroughly, and incubated at 4°C for 2 h. The precipitate was pelleted by centrifugation at 4,500 g for 5 min at 4°C, washed three times with pre-chilled acetone, and air-dried briefly. The protein pellet was redissolved in 200 mM triethylammonium bicarbonate (TEAB), dispersed by ultrasonication, and subjected to tryptic digestion.

### LC-MS/MS analysis

Tryptic peptides were dissolved in solvent A (0.1% formic acid in water, v/v) and directly loaded onto a homemade reversed-phase analytical column (15 cm length, 100 μm i.d.). Chromatographic separation was performed on a Vanquish Neo UHPLC system (ThermoFisher Scientific) using a gradient of solvent B (0.1% formic acid in 80% acetonitrile, v/v) at a constant flow rate of 450 nL/min. The gradient program was as follows: 0–14 min, 8%–27% B; 14–16 min, 27%–36% B; 16–17 min, 36%–99% B; 17–20 min, 99% B. Eluted peptides were ionized via a capillary source with an electrospray voltage of 1.6 kV and analyzed on a timsTOF HT mass spectrometer (Bruker). Data acquisition was performed in data-independent parallel accumulation serial fragmentation (dia-PASEF) mode. Full MS scans were acquired over an m/z range of 100–1700, and MS/MS scans were performed in the range of 425–1025 m/z with an isolation window of 25 m/z, collecting eight PASEF MS/MS scans per cycle.

### Database search and bioinformatic analysis

Raw DIA data were processed using Spectronaut (v.18) software. Tandem mass spectra were searched against the Mus_musculus_10090_SP_20231220.fasta database (17,191 entries) concatenated with a reverse decoy database. Trypsin/P was designated as the digestion enzyme, allowing up to two missed cleavages. Carbamidomethylation of cysteine was set as a fixed modification, while methionine oxidation and protein N-terminal acetylation were set as variable modifications. The false discovery rate (FDR) for proteins, peptides, and peptide-spectrum matches (PSMs) was controlled below 1%. Differentially expressed proteins (DEPs) were identified based on a threshold of P < 0.05 and fold-change ≥1.50 or ≤0.67. Subcellular localization analysis was performed using publicly available annotation tools. Functional enrichment analyses, including Gene Ontology (GO) classification, Kyoto Encyclopedia of Genes and Genomes (KEGG) pathway mapping, and WikiPathways analysis, were conducted to characterize the biological processes and signaling pathways significantly associated with the DEPs.

### Statistical Analysis

All data are presented as mean ± standard deviation (SD). Statistical analysis was performed using GraphPad Prism 9.0 software. Differences between two groups were analyzed using the two-tailed Student’s t-test. Comparisons among multiple groups were performed using one-way or two-way analysis of variance (ANOVA) followed by Tukey’s post-hoc test. A p-value < 0.05 was considered statistically significant.

## Results

### SIRT5 expression is downregulated in S-AKI

To investigate the potential role of SIRT5 in sepsis-induced AKI, we established a murine S-AKI model using CLP. As expected, the CLP group exhibited severe renal dysfunction, characterized by significantly elevated serum Cr and BUN levels compared to the Sham group (Fig 1A, 1B). Histological analysis via H&E staining revealed extensive tubular injury in the CLP group, including tubular dilation, cast formation, and brush border loss (Fig 1C).

**Fig 1:**
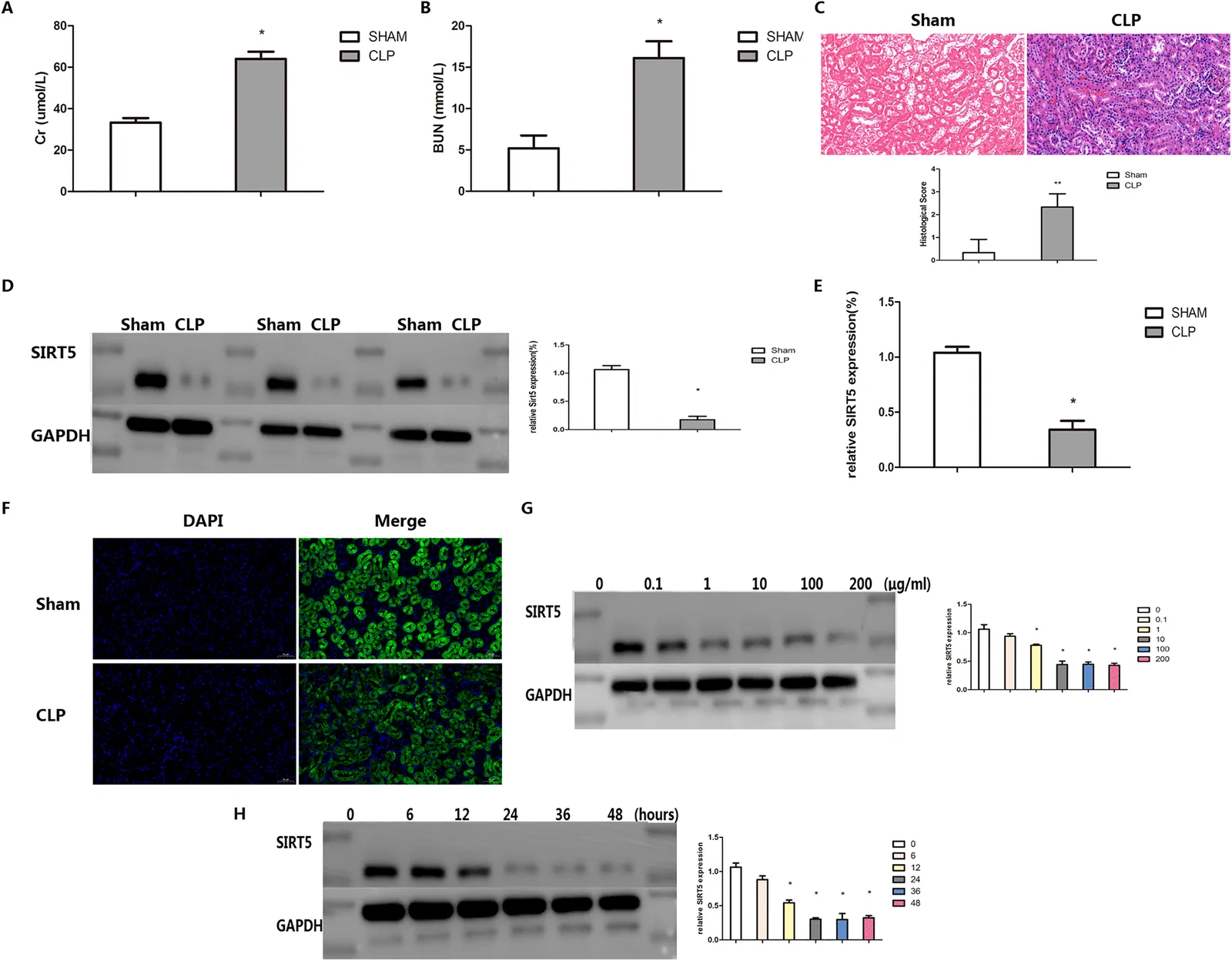
SIRT5 expression is downregulated in septic acute kidney injury (S-AKI). (A) Serum creatinine (Cr) levels in sham-operated and CLP-induced septic mice. (B) Blood urea nitrogen (BUN) levels in sham and CLP mice. (C) Representative H&E-stained kidney sections (scale bar = 50 μm) and tubular injury scores. CLP mice show tubular dilation, cast formation, and brush border loss. (D) Representative Western blots and quantification of SIRT5 protein expression in kidney tissues. (E) qRT-PCR analysis of SIRT5 mRNA levels. (F) Immunofluorescence staining of SIRT5 (green) and proximal tubule marker AQP1 (red) in kidney sections; nuclei stained with DAPI (blue). Scale bar = 20 μm. (G) Western blot showing dose-dependent downregulation of SIRT5 in HK-2 cells treated with increasing concentrations of LPS (0–200 μg/mL). (H) Time-dependent downregulation of SIRT5 in HK-2 cells treated with LPS (10 μg/mL) for indicated hours. Data are presented as mean ± SD. *p < 0.05, **p < 0.01 vs. sham or control; n = 5–6 per group.

We then examined SIRT5 expression in the kidney tissues. Both Western blot and qRT-PCR analyses demonstrated a marked reduction in SIRT5 protein and mRNA levels in the kidneys of CLP mice compared to Sham controls (Fig 1D, 1E). Immunofluorescence staining further confirmed that SIRT5 signal intensity was significantly diminished, particularly in the proximal tubules (AQP1-positive cells) (Fig 1F). Consistent with these in vivo findings, both Western blot analyses of HK-2 cells treated with LPS exhibited a dose- and time-dependent downregulation of SIRT5 expression (Fig 1G, 1H). These data suggest that the loss of SIRT5 is a pathological hallmark of S-AKI.

### Quantitative proteomic analysis reveals ferroptosis-associated pathways in S-AKI

To comprehensively define the renal proteomic alterations induced by sepsis, we performed quantitative proteomics profiling of kidney tissues from sham-operated and CLP-treated mice. The majority of identified peptide fragments ranged from 7 to 20 amino acids in length (Fig 2A), consistent with the expected distribution following tryptic digestion and mass spectrometric fragmentation. Assessment of the overall protein intensity distribution across all samples revealed minimal variation (Fig 2B), indicating high reproducibility of the proteomic data and suitability for reliable quantitative comparison.

**Fig 2:**
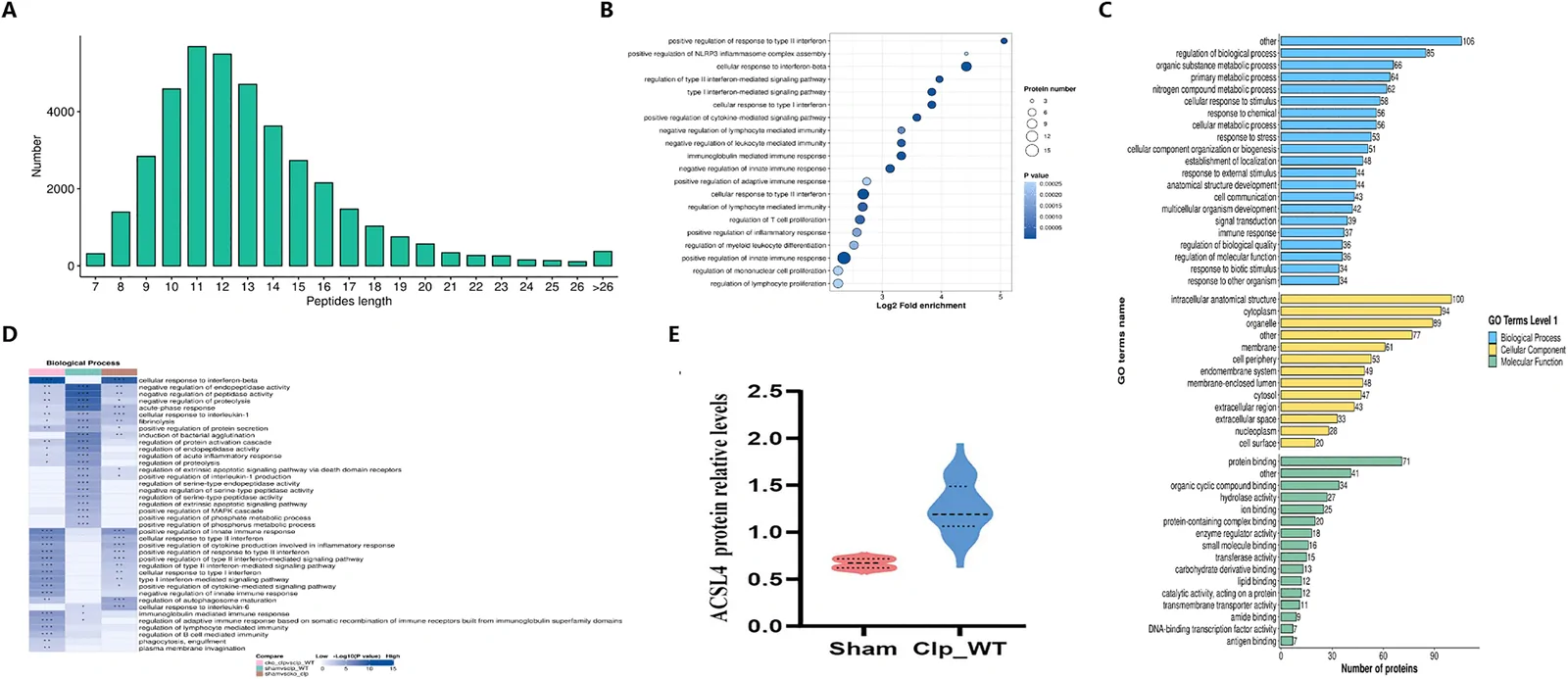
Quantitative proteomic analysis reveals ferroptosis-associated pathways in septic kidneys. (A) Distribution of identified peptide fragment lengths. (B) Protein intensity distribution across all samples shows high reproducibility. (C) Subcellular localization of differentially expressed proteins (DEPs). (D) KEGG and WikiPathways enrichment analyses showing significant enrichment of ferroptosis and iron homeostasis pathways. (E) Radar plot of representative DEPs, showing marked upregulation of ACSL4 and HMOX1 in CLP vs. sham group.n = 5 per group. DEPs defined by p < 0.05 and fold change ≥1.5 or ≤0.67.

Differentially expressed proteins (DEPs) were filtered using the criteria of P < 0.05 and a fold-change ≥ 1.50 (upregulated) or ≤ 0.67 (downregulated). Subcellular localization analysis indicated that the DEPs predominantly originated from the extracellular region (39.34%), cytoplasm (18.85%), and mitochondria (9.84%) (Fig 2C), suggesting that septic insult triggers widespread perturbations across multiple subcellular compartments, with notable involvement of mitochondrial proteins.

To gain insight into the biological significance of these proteomic changes, we performed functional enrichment analyses. GO enrichment analysis revealed that the DEPs were significantly enriched in biological processes such as the acute inflammatory response and cellular response to interleukin-1. Notably, KEGG pathway analysis revealed a significant enrichment of DEPs in the ferroptosis pathway, while WikiPathways analysis further highlighted the enrichment of iron homeostasis-related pathways (Fig 2D). These findings strongly suggest that disturbed iron metabolism and ferroptosis play a central role in sepsis-induced renal injury. Consistently, a radar plot of representative differential proteins demonstrated that the expression of ACSL4 and HMOX1 was markedly elevated in the CLP group compared to the Sham group (Fig 2E). Collectively, these proteomic results point to the prominent involvement of ferroptosis and oxidative stress in the pathogenesis of S-AKI, and identify ACSL4 as a significantly dysregulated ferroptosis driver in the septic kidney.

### SIRT5 overexpression attenuates S-AKI in vivo

To determine whether restoring SIRT5 confers renoprotection, we injected mice with rAAV9-SIRT5 to overexpress SIRT5 in the kidneys prior to CLP surgery. Successful overexpression was verified by Western blot (Fig 3A). Functional assessment showed that SIRT5 overexpression significantly blunted the CLP-induced elevation of serum Cr and BUN levels (Fig 3B, 3C). Histologically, kidneys from the SIRT5-overexpression group displayed preserved tubular architecture and significantly lower tubular injury scores compared to the CLP group (Fig 3D). Furthermore, TUNEL staining indicated that upregulation of SIRT5 significantly reduced the number of apoptotic cells in the renal cortex (Fig 3E). These findings indicate that SIRT5 alleviates sepsis-induced renal injury.

**Fig 3:**
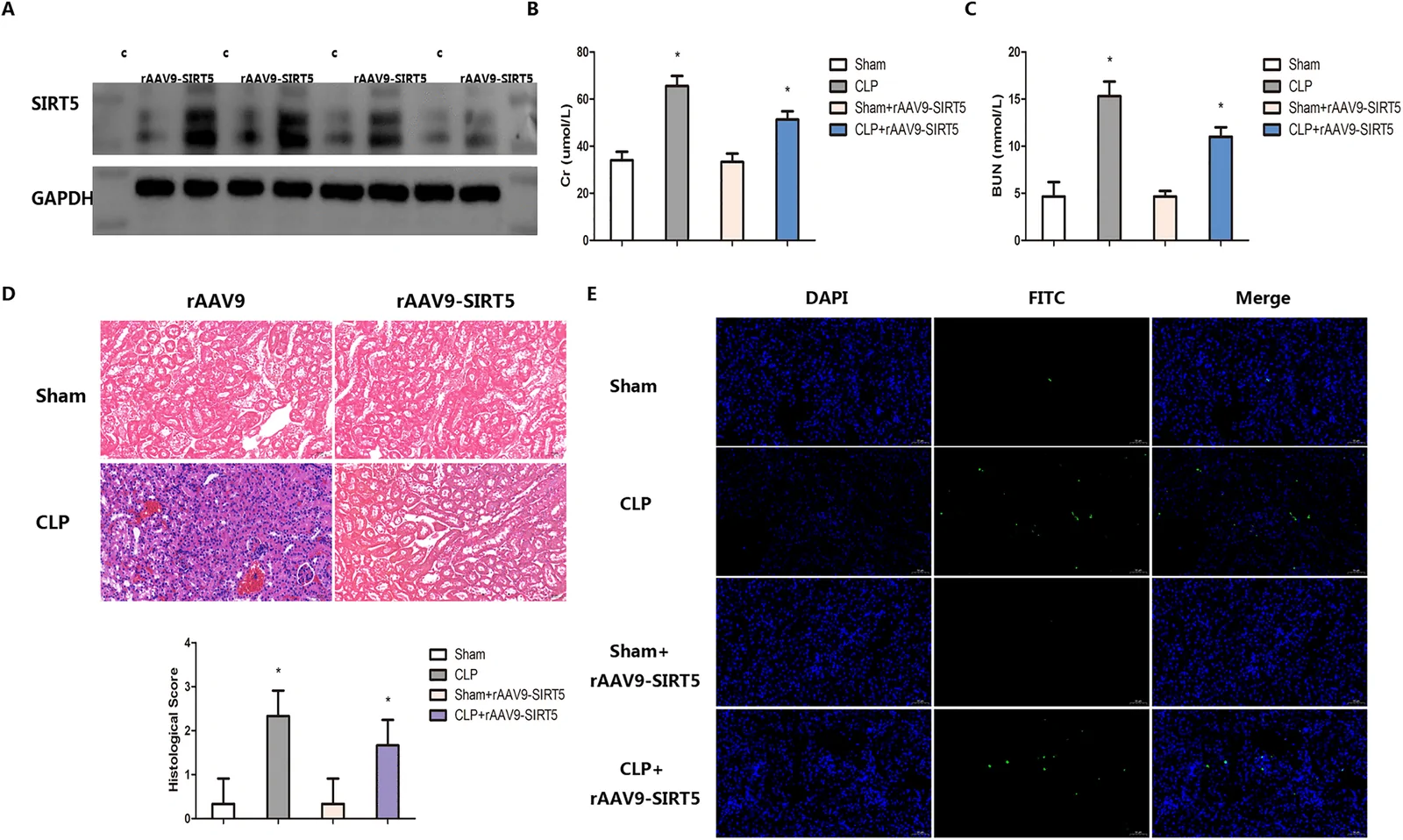
SIRT5 overexpression attenuates S-AKI in vivo. (A) Western blot validation of SIRT5 overexpression by rAAV9-SIRT5 in mouse kidneys. (B) Serum creatinine levels. (C) BUN levels. (D) Representative H&E-stained kidney sections and tubular injury scores. rAAV9-SIRT5 treatment preserves tubular architecture. (E) TUNEL staining (green) for apoptotic cells in renal cortex; nuclei counterstained with DAPI (blue). Quantification shows reduced apoptosis after SIRT5 overexpression. Data are mean ± SD. *p < 0.05, **p < 0.01; n = 6 per group. CLP, cecal ligation and puncture.

### SIRT5 deficiency exacerbates S-AKI in vivo

Opposite to the overexpression results, SIRT5 conditional knockout mice were generated (Fig 4A). Under CLP conditions, mice with SIRT5 deficiency exhibited worsened renal function, with even higher levels of Cr and BUN compared to the CLP group (Fig 4B, C). Pathological staining revealed aggravated tubular necrosis and more extensive lumen debris in the SIRT5 deficiency group (Fig 4D). Additionally, the expression of the kidney injury molecule KIM-1 was markedly higher in SIRT5 deficient mice (Fig 4E). Collectively, these results demonstrate that endogenous SIRT5 acts as a protective shield against septic kidney injury, and its deficiency worsens the pathological outcome.

**Fig 4:**
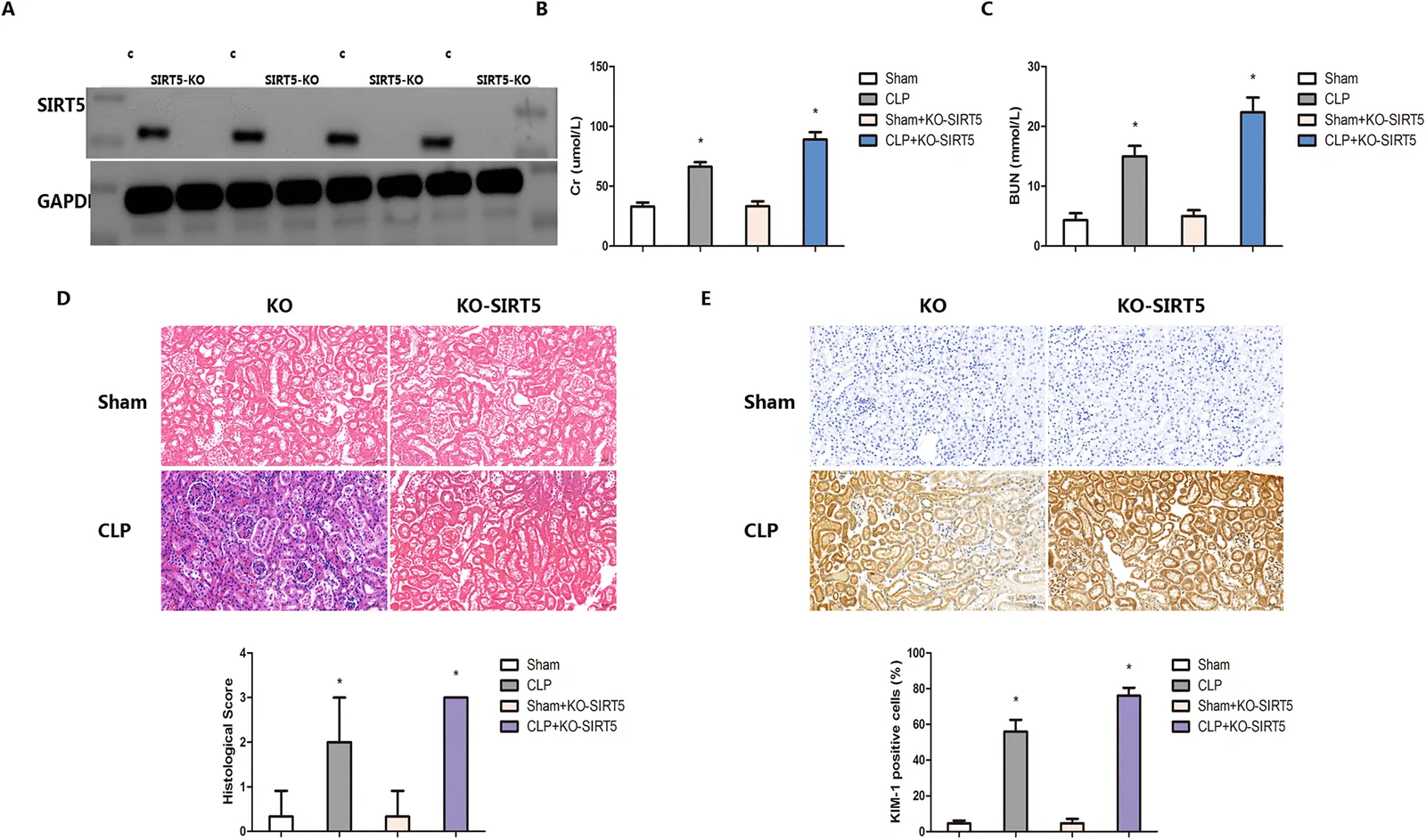
Renal tubule-specific SIRT5 deficiency exacerbates S-AKI. (A) Western blot confirming efficient SIRT5 knockout in renal tubules (SIRT5^flox/flox; Cdh16-Cre). (B) Serum creatinine levels. (C) BUN levels. (D) H&E staining and tubular injury scores showing aggravated injury in SIRT5-deficient mice after CLP. (E) Representative images and quantification of KIM-1-positive tubular cells. SIRT5 deficiency increases KIM-1 expression. Data are mean ± SD. *p < 0.05, **p < 0.01; n = 6 per group. KO, knockout.

### SIRT5 regulates mechanistic signs of ferroptosis

Given the emerging role of ferroptosis in S-AKI, we investigated whether SIRT5 regulates this process. Using TEM we observed that mitochondria in the renal tubular cells of CLP mice appeared shrunken with increased membrane density and vanished cristae. SIRT5 overexpression rescued these mitochondrial morphological defects, whereas SIRT5 deficiency exacerbated them (Fig 5A).

**Fig 5:**
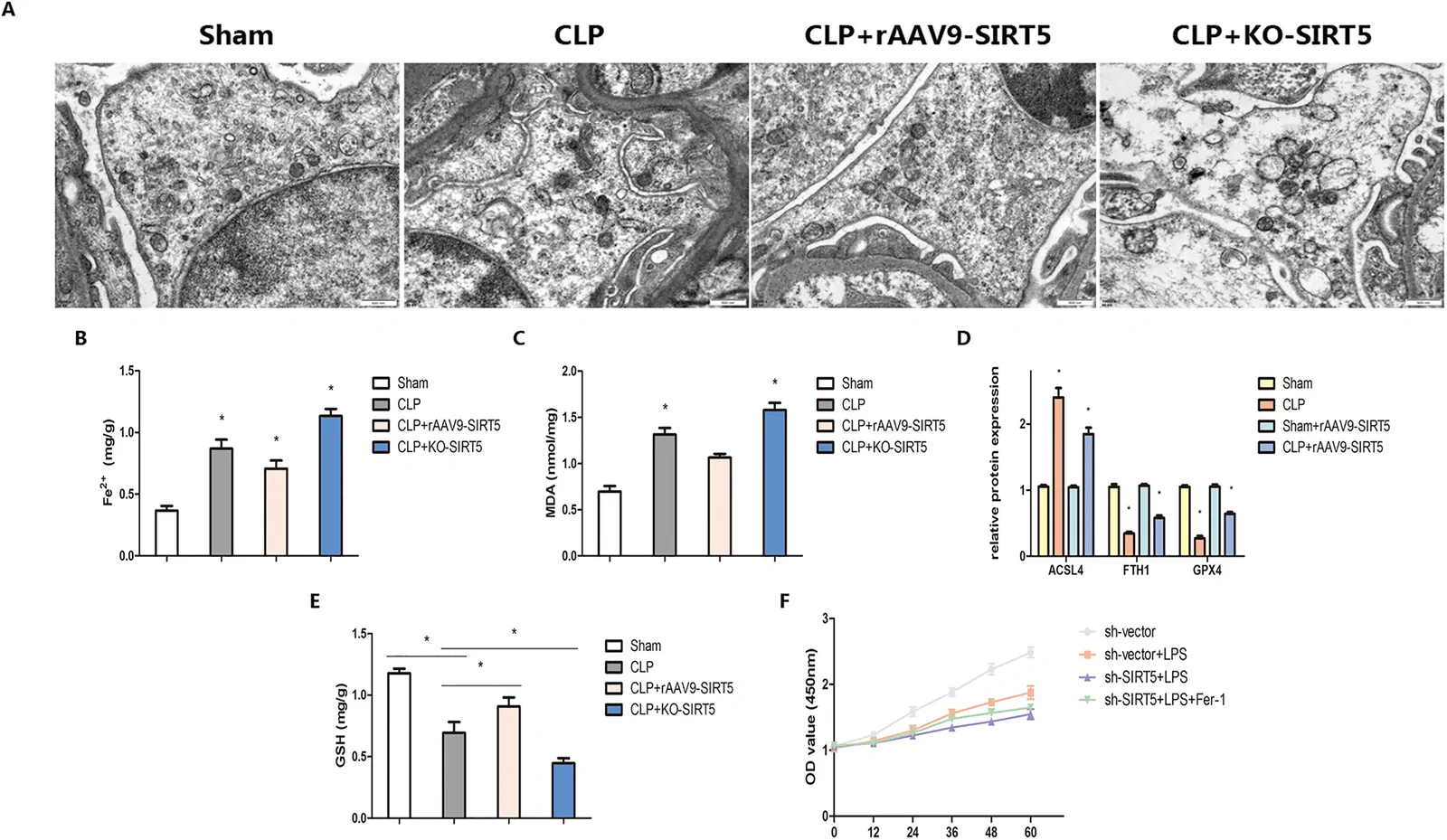
SIRT5 regulates ferroptosis in septic kidneys. (A) Transmission electron microscopy (TEM) images of renal tubular mitochondria. CLP induces mitochondrial shrinkage, increased membrane density, and cristae loss; SIRT5 overexpression rescues, while SIRT5 deficiency worsens defects. Scale bar = 0.5 μm. (B) Lipid ROS levels measured by DCFH-DA. (C) Malondialdehyde (MDA) levels. (D) Ferrous iron (Fe²⁺) levels. (E) Glutathione (GSH) levels. (F) Cell viability (CCK-8 assay) in HK-2 cells. Ferrostatin-1 (Fer-1, 1 μM) rescues the enhanced cell death caused by SIRT5 knockdown (sh-SIRT5) under LPS stimulation. Data are mean ± SD. *p < 0.05, **p < 0.01; n = 5–6 per group (in vivo) or 3 independent experiments (in vitro).

Biochemically, we measured key ferroptosis markers. CLP induction led to a significant accumulation of Lipid ROS, MDA and Fe^2+^, along with a depletion of the antioxidant GSH. SIRT5 overexpression significantly reversed these changes, reducing Fe^2+^, MDA and ROS levels while restoring GSH (Fig 5B-E). Conversely, SIRT5 deficiency further amplified oxidative stress and iron overload. In HK-2 cells, treatment with the ferroptosis inhibitor Ferrostatin-1 (Fer-1) abrogated the exacerbated cell death caused by SIRT5 knockdown (Fig 5F), confirming that SIRT5 protects against AKI primarily by suppressing ferroptosis.

### SIRT5 modulates the ACSL4/FTH1 axis

To elucidate the molecular mechanism by which SIRT5 inhibits ferroptosis, we analyzed the protein levels of key ferroptosis regulators. The Proteomics analysis were re-analyzed to assess the pathway mechanism of SIRT5 in AKI. The results showed that the ACSL4 signaling pathway was enriched (Fig 2E). ACSL4 signal is widely reported in AKI. Western blot analysis revealed that SIRT5 deficiency led to a significant upregulation of ACSL4 (a driver of ferroptosis) and a downregulation of FTH1 (the iron storage protein), while GPX4 levels were moderately suppressed (Fig 6A). Conversely, SIRT5 overexpression suppressed ACSL4 and restored FTH1 as well as GPX4 protein levels (Fig 6B).

**Fig 6:**
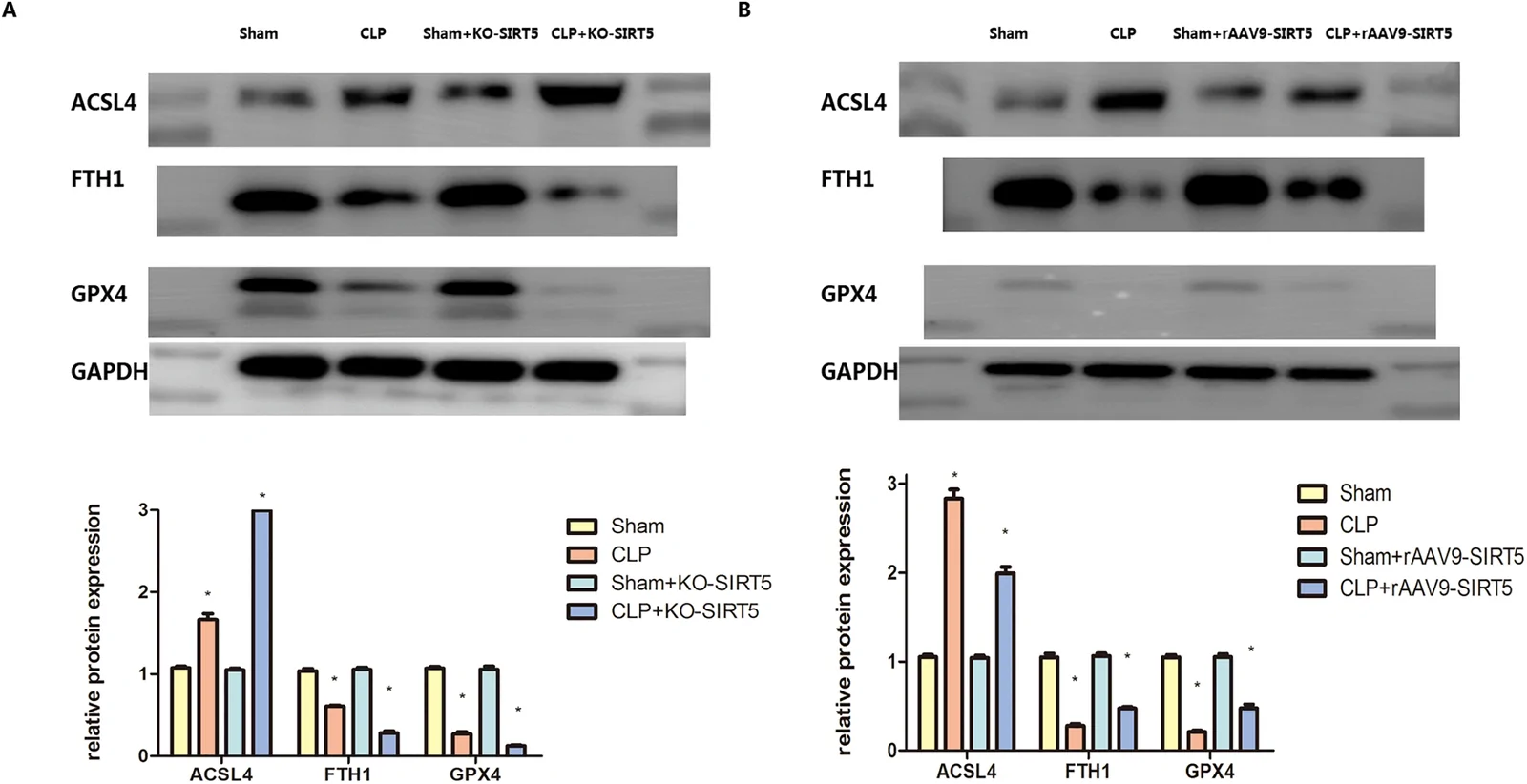
SIRT5 modulates the ACSL4/FTH1 axis. (A) Western blot analysis of ACSL4, FTH1, and GPX4 in kidney tissues from sham, CLP, and SIRT5-deficient (KO) mice. SIRT5 deficiency upregulates ACSL4 and downregulates FTH1 and GPX4. (B) Western blot analysis of the same proteins in kidneys from sham, CLP, and SIRT5-overexpressing (rAAV9-SIRT5) mice. SIRT5 overexpression reverses these changes. Quantification of protein levels is shown below the blots. Data are mean ± SD. *p < 0.05, **p < 0.01; n = 4 per group.

### Knockdown of ACSL4 rescues the injury induced by SIRT5 deficiency

To confirm that the ACSL4/FTH1 axis is the functional effector downstream of SIRT5, we performed rescue experiments in HK-2 cells. We established a “SIRT5-Deficient” model (using shSIRT5) which exhibited high ACSL4 levels and severe ferroptosis upon LPS stimulation. We then co-transfected these cells with shACSL4 (Fig 7A).

**Fig 7:**
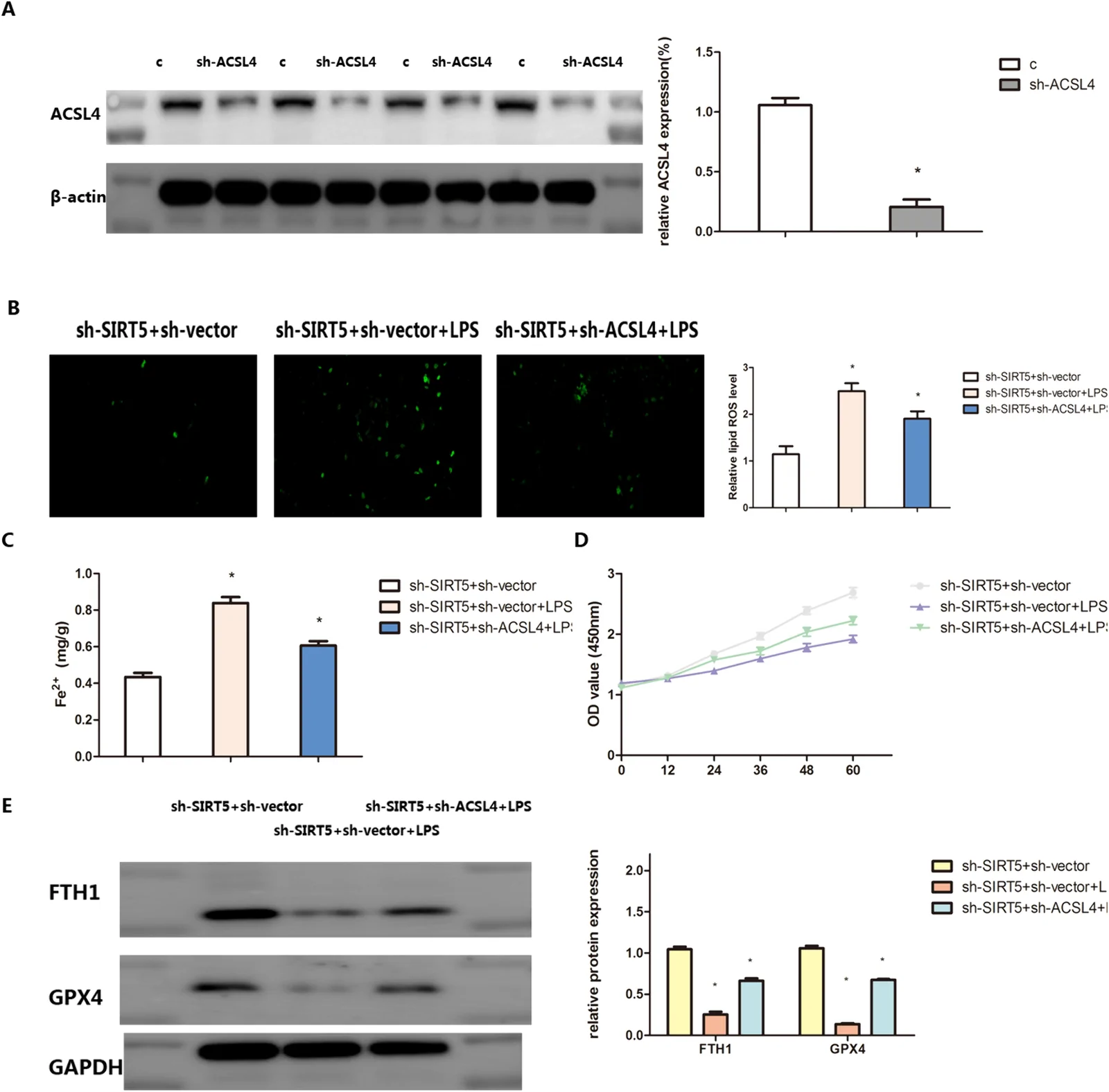
Knockdown of ACSL4 rescues SIRT5 deficiency-induced ferroptosis and cell injury. (A) Validation of ACSL4 knockdown by shRNA in HK-2 cells (Western blot). (B) Relative ROS levels. SIRT5 deficiency (sh-SIRT5) increases ROS; co-knockdown of ACSL4 (sh-SIRT5 + sh-ACSL4) reduces ROS. (C) Intracellular Fe² ⁺ levels. (D) Cell viability (CCK-8). ACSL4 knockdown restores viability in SIRT5-deficient cells treated with LPS. (E) Western blot showing that ACSL4 knockdown partially restores FTH1 and GPX4 expression in SIRT5-deficient cells. Data are mean ± SD of three independent experiments. *p < 0.05, **p < 0.01; ns, not significant.

Recentering the axis, the knockdown of ACSL4 in the SIRT5 deficient background significantly reduced lipid peroxidationand intracellular iron accumulation compared to the shSIRT5 alone group (Fig 7B, C). Furthermore, CCK-8 showed that knockdown of ACSL4 abrogated the increased cell death caused by SIRT5 deficiency (Fig 7D). Western blot confirmed that shACSL4 treatment successfully blocked the downstream ferroptotic machinery (Fig 7E). These data provide conclusive evidence that SIRT5 exerts its renoprotective effects by restraining ACSL4-dependent ferroptosis.

### SIRT5 deficiency disrupts the ACSL4/FTH1 axis to drive ferroptosis in S‑AKI

The mechanistic framework of this study is summarized in the graphical abstract (Fig 8). Taken together, our data support a model in which sepsis induces SIRT5 downregulation in renal tubular epithelial cells, leading to dysregulation of the ACSL4/FTH1 axis. Increased ACSL4 promotes membrane lipid remodeling and enhances susceptibility to lipid peroxidation, whereas reduced FTH1 weakens iron sequestration and expands the labile iron pool. In parallel, impaired antioxidant defense further amplifies lipid peroxide accumulation, ultimately resulting in ferroptotic cell death and tubular injury. Restoration of SIRT5 reverses these changes and protects against septic kidney damage.

**Fig 8:**
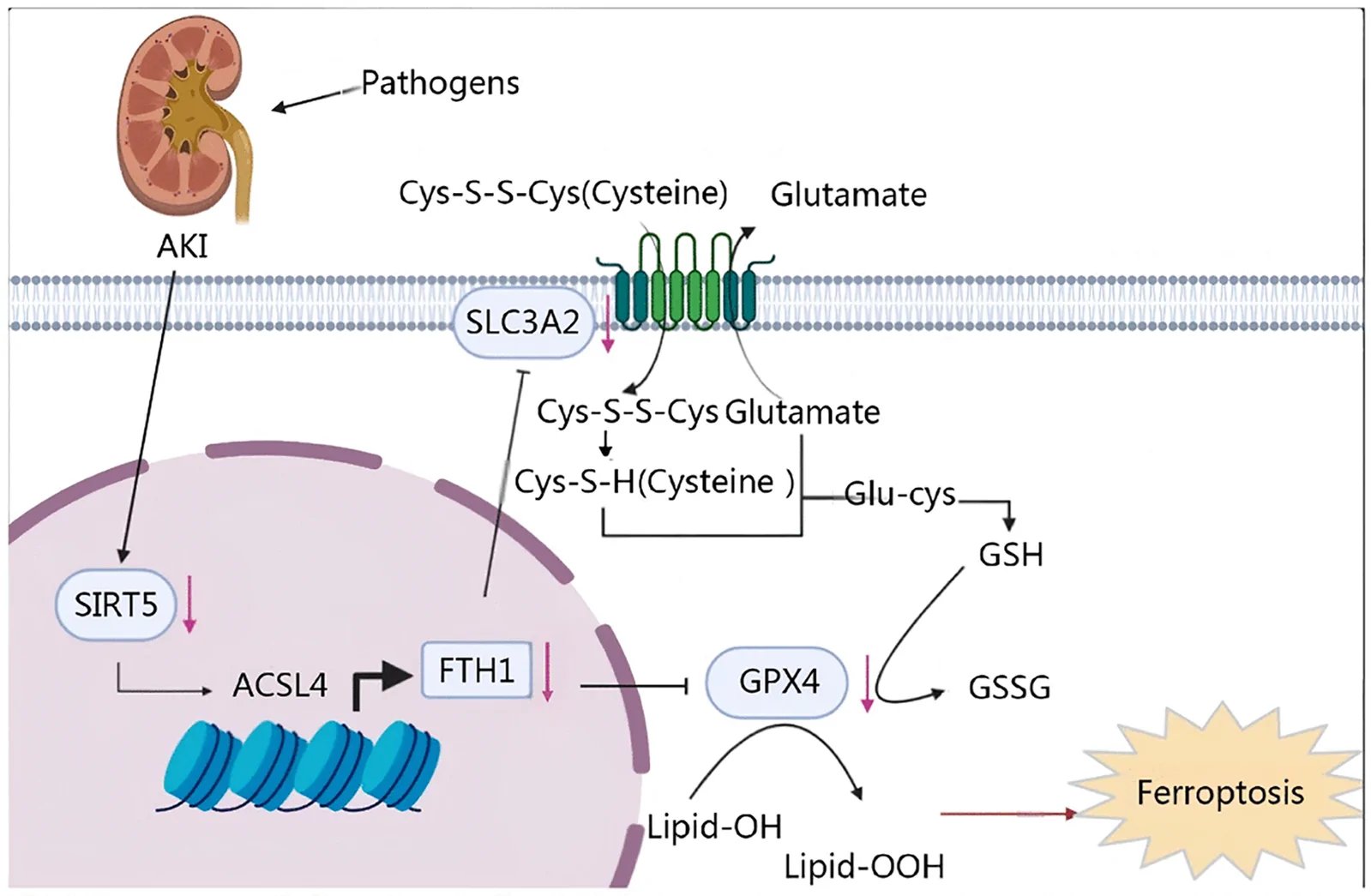
SIRT5 deficiency exacerbates ferroptosis in S-AKI by activating the ACSL4/FTH1 axis.

## Discussion

SIRT5 is a mitochondrial NAD ⁺ -dependent desuccinylase that plays an important role in maintaining mitochondrial metabolic homeostasis and cellular stress responses. Accumulating evidence suggests that SIRT5 exerts protective effects in multiple pathological conditions, including cardiovascular injury, neurodegeneration [19,20], and renal injury [21,22]. However, its role in sepsis-associated acute kidney injury (S-AKI) has remained largely undefined. In the present study, we demonstrated that SIRT5 expression was significantly reduced in both septic kidney tissues and LPS-treated proximal tubular epithelial cells. Functional gain- and loss-of-function experiments further showed that SIRT5 deficiency aggravated renal dysfunction and tubular injury, whereas SIRT5 overexpression alleviated sepsis-induced kidney damage. These findings identify SIRT5 as an endogenous protective factor in S-AKI.

A growing body of evidence has recognized ferroptosis as an important contributor to the pathogenesis of AKI, including S-AKI [23–25]. Unlike apoptosis or necroptosis, ferroptosis is characterized by iron-dependent lipid peroxidation, mitochondrial shrinkage, and depletion of antioxidant defenses. In this study, quantitative proteomic analysis of septic kidneys revealed significant enrichment of ferroptosis-related and iron homeostasis pathways, suggesting that disordered redox and iron metabolism are central features of S-AKI. Consistent with this notion, we found that CLP-induced sepsis caused typical ferroptotic changes in renal tubular cells, including increased lipid ROS, elevated malondialdehyde, iron accumulation, GSH depletion, and mitochondrial structural damage. Importantly, these changes were further exacerbated by SIRT5 deficiency and attenuated by SIRT5 overexpression or ferrostatin-1 treatment, indicating that SIRT5 suppresses tubular ferroptosis during sepsis.

A recent study reported that SIRT5 suppresses ferroptosis in S-AKI through desuccinylation of PRDX6 [26], identifying post-translational modification of an antioxidant enzyme as a key protective mechanism. Our findings extend this understanding by revealing a distinct and complementary pathway: SIRT5 deficiency dysregulates the ACSL4/FTH1 axis, promoting ferroptosis through enhanced lipid peroxidation and impaired iron sequestration. Together, these studies suggest that SIRT5 may exert multifaceted protection against ferroptosis in septic kidneys—acting both via direct modification of antioxidant proteins (PRDX6) and via regulation of the core ferroptotic machinery (ACSL4/FTH1).

Mechanistically, our data suggest that SIRT5 regulates ferroptotic susceptibility through the ACSL4/FTH1 axis. ACSL4 is a key determinant of ferroptosis sensitivity by facilitating the incorporation of polyunsaturated fatty acids into membrane phospholipids, thereby increasing the substrate availability for lipid peroxidation [27–29]. In contrast, FTH1 serves as a major intracellular iron-storage protein that limits the labile iron pool and protects cells from iron-catalyzed oxidative damage [30,31]. We found that SIRT5 deficiency led to upregulation of ACSL4 and downregulation of FTH1, accompanied by suppression of GPX4, thereby creating a cellular environment favoring ferroptosis. Conversely, SIRT5 overexpression reversed these changes. More importantly, knockdown of ACSL4 in SIRT5-deficient HK-2 cells reduced lipid peroxidation, iron overload, and cell death, confirming that ACSL4 is a critical downstream effector in SIRT5-mediated protection against ferroptosis. These results support a model in which SIRT5 maintains redox and iron homeostasis in renal tubular cells by restraining ACSL4-driven lipid remodeling and preserving FTH1-dependent iron sequestration [32,33].

S-AKI remains a clinical challenge with high mortality and a lack of targeted pharmacological interventions [34,35]. The present study also provides a broader perspective on the interplay between mitochondrial dysfunction and ferroptosis in septic kidney injury. Because SIRT5 is predominantly localized in mitochondria, its protective effects may be closely linked to mitochondrial quality control, bioenergetic regulation, and oxidative stress adaptation. Although our study did not directly define whether SIRT5 modulates ACSL4 or FTH1 through direct post-translational modification, the observed changes in these proteins indicate that SIRT5 influences ferroptosis-related signaling at multiple levels. Future studies are warranted to determine whether SIRT5 directly desuccinylates components of the ferroptosis machinery or acts through upstream metabolic pathways that affect protein stability and expression.

Clinically, S-AKI remains a major complication of sepsis and is associated with high mortality, prolonged hospitalization, and increased risk of chronic kidney disease. Current therapies are largely supportive and do not directly target the molecular mechanisms driving tubular injury. Our findings suggest that restoring SIRT5 activity may represent a promising therapeutic strategy for S-AKI. In addition, targeting the ACSL4/FTH1 axis or suppressing ferroptosis may provide alternative approaches to mitigate septic renal injury. Although pharmacological activators of SIRT5 are still limited, the present study provides a rationale for future development of SIRT5-based interventions in septic kidney disease.

Our experimental results show that: 1. SIRT5 is highly expressed in normal kidneys but is significantly downregulated in S-AKI in both in vivo and in vitro models; 2. SIRT5 deficiency disrupts the ACSL4/FTH1 axis, leading to FTH1 depletion and ACSL4 accumulation, which triggers uncontrolled lipid peroxidation and ferroptosis; 3. Restoring SIRT5 expression or blocking the downstream ferroptotic pathway effectively attenuates renal injury and preserves kidney function during sepsis; 4.The TEM analysis in this study was primarily qualitative. Although the morphological changes observed were consistent and representative, quantitative morphometric parameters such as cristae density and mitochondrial membrane density were not measured. Future studies incorporating quantitative TEM analysis will help further substantiate these findings.

Several limitations of this study should be acknowledged. First, the precise molecular mechanism by which SIRT5 regulates ACSL4 and FTH1 remains to be clarified. Whether this regulation occurs through direct deacylation, altered transcriptional control, or secondary metabolic remodeling was not addressed in the present work. Second, although ferroptosis was identified as a major mode of cell death in this model, other forms of regulated cell death, including apoptosis, pyroptosis, and necroptosis, may also contribute to S-AKI and may interact with ferroptotic signaling. Third, the present study relied mainly on murine and cell culture models; therefore, validation in human clinical samples will be necessary to assess the translational relevance of the SIRT5-ACSL4/FTH1 axis.

In summary, utilizing both genetic and pharmacological approaches, we have elucidated the essential protective role of SIRT5 in S-AKI. SIRT5 maintains the homeostasis of the ACSL4/FTH1 axis, preventing the onset of lethal ferroptosis in renal tubular cells. The loss of this protective mechanism during sepsis accelerates kidney injury, highlighting the SIRT5/ACSL4/FTH1 axis as a vital target for therapeutic intervention.

SIRT5 prevents sepsis-induced renal injury by maintaining the balance of the ACSL4/FTH1 axis. SIRT5 deficiency leads to the downregulation of FTH1 and the upregulation of ACSL4, thereby triggering an iron-dependent ferroptosis cascade that exacerbates AKI.

## Supporting information

S1 Raw Images Original uncropped and unadjusted Western blot images. Full-length membranes corresponding to Figures 1D, 1G, 1H, 3A, 4A, 6A, 6B, 7A, and 7E, with cropped regions indicated.
(DOCX)
