# Supplementary material for: SIRT5 deficiency exacerbates sepsis acute renal injury by activating ACSL4/FTH1 axis dependent ferroptosis
Source: PLoS One. 2026 Oct 27;21(10):e0361332. doi: 10.1371/journal.pone.0361332 (PMC13712530; doi:10.1371/journal.pone.0361332)
Supplement: Supplementary file 1 [file pone.0361332.s001.docx]

1D

1D

1G

1G

1H

1H

3A

3A

4A

4A

6A-ACSL4

6A-FTH1

6A-GPX4

6A-GAPDH

6B-ACSL4

6B-FTH1

6B-GPX4

6B-GAPDH

7A

7A

7E-FTH1

7E-GPX4

7E-GAPDH
