# Supplementary material for: SIRT5 deficiency exacerbates sepsis acute renal injury by activating ACSL4/FTH1 axis dependent ferroptosis
Source: PLoS One. 2026 Oct 27;21(10):e0361332. doi: 10.1371/journal.pone.0361332 (PMC13712530; doi:10.1371/journal.pone.0361332)
Supplement: Supplementary file 2 [file pone.0361332.s002.docx]

Journal Requirements:

When submitting your revision, we need you to address these additional requirements.

1.Please ensure that your manuscript meets PLOS ONE's style requirements, including those for file naming. The PLOS ONE style templates can be found at

https://journals.plos.org/plosone/s/file?id=wjVg/PLOSOne_formatting_sample_main_body.pdf and

[https://journals.plos.org/plosone/s/file?id=ba62/PLOSOne_formatting_sample_title_authors_affiliations.pdf](https://mailgw.whu.edu.cn/linkserver?dest=aHR0cHM6Ly90cmFjay5lZGl0b3JpYWxtYW5hZ2VyLmNvbS9DTDAvaHR0cHM6JTJGJTJGam91cm5h%0D%0AbHMucGxvcy5vcmclMkZwbG9zb25lJTJGcyUyRmZpbGUlM0ZpZD1iYTYyJTJGUExPU09uZV9mb3Jt%0D%0AYXR0aW5nX3NhbXBsZV90aXRsZV9hdXRob3JzX2FmZmlsaWF0aW9ucy5wZGYvMS8wMTBmMDE5ZjY4%0D%0AN2Q1NTY1LTQ3NmRlNjFkLWJjZjktNDY4OC04NGVkLTU4NjFmYjdhMDk0YS0wMDAwMDAvMVJyQ2lY%0D%0AM3RkWjBJaUNTYWc3Tk9OLU96cjV3VnMxSzNPLWg3dlJkMEdWMD0yNTg=%0D%0A&tid=_____wC3Jth8MFhq645mAA--.7S2&rcpt=yuxi2018@whu.edu.cn&ifnotice=1&rindex=3)

We thank the reviewer for this reminder. We have carefully reviewed the PLOS ONE style requirements and formatting templates, and have revised our manuscript accordingly.

2. We note that the grant information you provided in the ‘Funding Information’ and ‘Financial Disclosure’ sections do not match.

When you resubmit, please ensure that you provide the correct grant numbers for the awards you received for your study in the ‘Funding Information’ section.

We thank the editor for bringing this discrepancy to our attention. We have carefully reviewed both the 'Funding Information' section in the submission system and the financial disclosure statement in the manuscript, and have now corrected them so that they are fully consistent.

3. Thank you for stating the following financial disclosure:

“Hubei Provincial Natural Science Foundation Joint Fund Program(2025AFD830)”

Please state what role the funders took in the study.  If the funders had no role, please state: "The funders had no role in study design, data collection and analysis, decision to publish, or preparation of the manuscript."

If this statement is not correct you must amend it as needed.

Please include this amended Role of Funder statement in your cover letter; we will change the online submission form on your behalf.

This research was funded by Hubei Provincial Natural Science Foundation Joint Fund Program(2025AFD830); and the Hubei Provincial Natural Science Foundation (2024AFC041). The funders had no role in study design, data collection and analysis, decision to publish, or preparation of the manuscript.

4. We note that your Data Availability Statement is currently as follows: “Data is provided within the manuscript or supplementary information files.”

Please confirm at this time whether or not your submission contains all raw data required to replicate the results of your study. Authors must share the “minimal data set” for their submission. PLOS defines the minimal data set to consist of the data required to replicate all study findings reported in the article, as well as related metadata and methods (https://journals.plos.org/plosone/s/data-availability#loc-minimal-data-set-definition).

For example, authors should submit the following data:

- The values behind the means, standard deviations and other measures reported;

- The values used to build graphs;

- The points extracted from images for analysis.

Authors do not need to submit their entire data set if only a portion of the data was used in the reported study.

If your submission does not contain these data, please either upload them as Supporting Information files or deposit them to a stable, public repository and provide us with the relevant URLs, DOIs, or accession numbers. For a list of recommended repositories, please see https://journals.plos.org/plosone/s/recommended-repositories.

If there are ethical or legal restrictions on sharing a de-identified data set, please explain them in detail (e.g., data contain potentially sensitive information, data are owned by a third-party organization, etc.) and who has imposed them (e.g., an ethics committee). Please also provide contact information for a data access committee, ethics committee, or other institutional body to which data requests may be sent. If data are owned by a third party, please indicate how others may request data access.

OK, I see. Thank you.

5. We notice that your supplementary figures are uploaded with the file type 'Figure'. Please amend the file type to 'Supporting Information'. Please ensure that each Supporting Information file has a legend listed in the manuscript after the references list.

Thank you for pointing this out. We have corrected the file type of all supplementary figures from "Figure" to "Supporting Information" in the submission system. Additionally, we have added a complete "Supporting Information" section listing legends for all supplementary files after the references list in the revised manuscript. We hope these changes meet the journal's requirements.

6. PLOS ONE now requires that authors provide the original uncropped and unadjusted images underlying all blot or gel results reported in a submission’s figures or Supporting Information files. This policy and the journal’s other requirements for blot/gel reporting and figure preparation are described in detail at https://journals.plos.org/plosone/s/figures#loc-blot-and-gel-reporting-requirements and https://journals.plos.org/plosone/s/figures#loc-preparing-figures-from-image-files. When you submit your revised manuscript, please ensure that your figures adhere fully to these guidelines and provide the original underlying images for all blot or gel data reported in your submission. See the following link for instructions on providing the original image data: https://journals.plos.org/plosone/s/figures#loc-original-images-for-blots-and-gels.

In your cover letter, please note whether your blot/gel image data are in Supporting Information or posted at a public data repository, provide the repository URL if relevant, and provide specific details as to which raw blot/gel images, if any, are not available. Email us at [plosone@plos.org](mailto:plosone@plos.org) if you have any questions.

In accordance with the journal's updated policy on blot/gel reporting and figure preparation, we have carefully reviewed all of our figures and have ensured that they comply with the guidelines. The original, uncropped, and unadjusted blot/gel images underlying all figures have been provided as Supporting Information (S1 Raw Images) in the revised submission. These raw images are clearly labeled with the corresponding figure numbers and molecular weight markers.

We confirm that no blot or gel images have been manipulated in any way that misrepresents the data. All images are available in their full-length format, with the cropped regions used in the main figures clearly indicated. No raw blot/gel images are unavailable.

6. If the reviewer comments include a recommendation to cite specific previously published works, please review and evaluate these publications to determine whether they are relevant and should be cited. There is no requirement to cite these works unless the editor has indicated otherwise.

We have carefully reviewed this work and found it highly relevant to our study. Accordingly, we have incorporated this reference into the revised manuscript, where it appropriately supports our discussion.

We thank the reviewer for this valuable suggestion.

[Note: HTML markup is below. Please do not edit.]

Reviewer's Responses to Questions

**Comments to the Author**

1. Is the manuscript technically sound, and do the data support the conclusions?

The manuscript must describe a technically sound piece of scientific research with data that supports the conclusions. Experiments must have been conducted rigorously, with appropriate controls, replication, and sample sizes. The conclusions must be drawn appropriately based on the data presented.

Reviewer #1: Yes

Reviewer #2: Yes

2. Has the statistical analysis been performed appropriately and rigorously?

Reviewer #1: Yes

Reviewer #2: Yes

3. Have the authors made all data underlying the findings in their manuscript fully available?

The [PLOS Data policy](https://mailgw.whu.edu.cn/linkserver?dest=aHR0cDovL3RyYWNrLmVkaXRvcmlhbG1hbmFnZXIuY29tL0NMMC9odHRwOiUyRiUyRnd3dy5wbG9z%0D%0Ab25lLm9yZyUyRnN0YXRpYyUyRnBvbGljaWVzLmFjdGlvbiUyM3NoYXJpbmcvMS8wMTBmMDE5ZjY4%0D%0AN2Q1NTY1LTQ3NmRlNjFkLWJjZjktNDY4OC04NGVkLTU4NjFmYjdhMDk0YS0wMDAwMDAvQWdjd2Zl%0D%0AUWJLWktxVnk4MHRXQmQyY2UyV3FnaS1pUzFTUEVoTzU5SGw4dz0yNTg=%0D%0A&tid=_____wC3Jth8MFhq645mAA--.7S2&rcpt=yuxi2018@whu.edu.cn&ifnotice=1&rindex=4) requires authors to make all data underlying the findings described in their manuscript fully available without restriction, with rare exception (please refer to the Data Availability Statement in the manuscript PDF file). The data should be provided as part of the manuscript or its supporting information, or deposited to a public repository. For example, in addition to summary statistics, the data points behind means, medians and variance measures should be available. If there are restrictions on publicly sharing data—e.g. participant privacy or use of data from a third party—those must be specified.

Reviewer #1: Yes

Reviewer #2: Yes

4. Is the manuscript presented in an intelligible fashion and written in standard English?

PLOS ONE does not copyedit accepted manuscripts, so the language in submitted articles must be clear, correct, and unambiguous. Any typographical or grammatical errors should be corrected at revision, so please note any specific errors here.

Reviewer #1: Yes

Reviewer #2: Yes

5. Review Comments to the Author

Please use the space provided to explain your answers to the questions above. You may also include additional comments for the author, including concerns about dual publication, research ethics, or publication ethics. (Please upload your review as an attachment if it exceeds 20,000 characters)

Reviewer #1: 1. In the Results section under "SIRT5 expression is downregulated in S-AKI," "BNU" appears to be a typo and should be corrected to "BUN."

We thank the reviewer for pointing out this typographical error. We have corrected "BNU" to "BUN" in the Results section. We have also carefully checked the entire manuscript for similar errors.

2. For the TEM data, if possible, include quantitative analysis of the observed mitochondrial alterations. Measurements such as cristae density, mitochondrial membrane density, and other morphological parameters would strengthen the findings.

 We sincerely appreciate the reviewer's constructive suggestion. We fully agree that quantitative morphometric analysis would further strengthen the TEM findings. However, due to the limited number of remaining TEM grids, we are unable to perform comprehensive quantitative measurements at this stage. We have therefore added this point as a limitation in the Discussion section and will include quantitative mitochondrial morphological analysis in our future studies.

3. For Figure 1C, 3D, 4D. Please provide a more detailed description of the histological scoring method, including how the score was determined and the criteria used for evaluation.

We thank the reviewer for this suggestion. We have now provided a detailed description of the histological scoring method in the Methods section.

4. Please provide the full, uncropped, and unedited Western blot images from which the quantification was derived, preferably as supplementary material.

We agree with the reviewer's request. In compliance with PLOS ONE's blot/gel reporting policy, we have compiled all original, uncropped, and unedited Western blot images underlying our quantification. These are now provided as S1 Raw Images in the revised submission.

Reviewer #2: The author of the manuscript investigated the contribution of SIRT5 in sepsis‑induced renal injury by employing several in vivo and in vitro experimental approaches. They focused on the correlation between SIRT5 and ferroptosis, particularly on the ACSL4/FTH1 axis. The manuscript could be of interest; however, I identified several major issues that should be addressed before publication.

Major:

1. The abstract is chaotic. I suggest rewriting it in a more rational and coherent manner.

We fully agree with the reviewer that the original abstract was poorly organized and lacked logical flow. We have therefore completely rewritten the abstract to present our findings in a more coherent and systematic manner. The revised abstract now follows a clear structure: background and knowledge gap → key experimental models → major findings → mechanistic insights → functional validation → conclusion and translational significance. We have also streamlined the methodological descriptions to avoid unnecessary technical details, and have emphasized the core mechanistic discovery (the SIRT5–ACSL4/FTH1 axis in ferroptosis suppression) throughout. We believe the revised abstract now reads more logically and conveys the scientific narrative more effectively.

2. In the introduction, the authors state that the relationship between SIRT5 and ferroptosis is unknown. Nevertheless, another paper published in Redox Report (PMID: 41960638) demonstrated the key role of SIRT5 in protecting the kidney against injury and its involvement in ferroptosis. I did not find this citation in the reference list. Therefore, I suggest inserting this article in the Introduction section and/or in the Discussion, in order to better contextualize the originality of the present study.

We sincerely thank the reviewer for bringing this important publication to our attention. We have now carefully read the paper by Lin et al. (Redox Report, 2026, PMID: 41960638), which demonstrates that SIRT5 protects against sepsis‑associated AKI by desuccinylating PRDX6 at lysine 209, thereby suppressing ferroptosis. We have now cited this paper in both the Discussion sections of the revised manuscript.

3. It is not clear whether approval no. 20250205A refers only to the SIRT5‑overexpression protocol or also to the SIRT5‑KO model. In general, the methodological section requires substantial revision. The authors used a wide range of experimental approaches, but these are not clearly or systematically described.

We sincerely thank the reviewer for this critical and constructive comment. We fully agree that the methodological description in our original submission was insufficiently clear and systematic. we have carefully reviewed and revised the descriptions of each experimental method to ensure they are described in sufficient detail and in a logical sequence that follows the chronological flow of the experiments. We believe these revisions substantially improve the clarity, reproducibility, and readability of the Methods section. All changes are highlighted in the revised manuscript. We are grateful to the reviewer for prompting us to make these important improvements.The Methods section has been restructured to follow a logical flow progressing from in vivo experiments to in vitro studies, then through histological, biochemical, protein-level, and proteomic analyses, concluding with statistical evaluation. This reorganization renders the methodological descriptions more systematic and reader-friendly.

4. In the Results section, the authors report a “murine S‑AKI model using CLP,” but the manuscript does not clearly describe how sepsis was induced. This information should be explicitly included in the Methods.

We thank the reviewer for pointing out this omission. In the revised Methods section, we have added a dedicated subsection which now provides a complete and step‑by‑step description of the CLP procedure. Specifically, we have included details on anesthesia (isoflurane inhalation), surgical incision, cecal ligation (approximately 50% of cecal length with 4‑0 silk suture), cecal puncture (twice with a 22‑gauge needle), postoperative fluid resuscitation (1 mL pre‑warmed normal saline, subcutaneously), and the time point of sample collection (24 h after surgery). The sham operation is also clearly defined. We believe these additions now provide sufficient detail for reproducibility of the model.

5.The graphs in the figures are not easily readable. I recommend improving their resolution, layout, and clarity.

We thank the reviewer for this helpful suggestion. We have carefully revised all figures to improve their resolution, layout, and overall clarity.

6. PLOS authors have the option to publish the peer review history of their article ([what does this mean?](https://mailgw.whu.edu.cn/linkserver?dest=aHR0cHM6Ly90cmFjay5lZGl0b3JpYWxtYW5hZ2VyLmNvbS9DTDAvaHR0cHM6JTJGJTJGam91cm5h%0D%0AbHMucGxvcy5vcmclMkZwbG9zb25lJTJGcyUyRmVkaXRvcmlhbC1hbmQtcGVlci1yZXZpZXctcHJv%0D%0AY2VzcyUyM2xvYy1wZWVyLXJldmlldy1oaXN0b3J5LzEvMDEwZjAxOWY2ODdkNTU2NS00NzZkZTYx%0D%0AZC1iY2Y5LTQ2ODgtODRlZC01ODYxZmI3YTA5NGEtMDAwMDAwL1RnaFV6LW9xNmxoTjlxSkJwRHlT%0D%0AQWMwS3JkaFdEWTZaZWRWMnlXNm12YVk9MjU4%0D%0A&tid=_____wC3Jth8MFhq645mAA--.7S2&rcpt=yuxi2018@whu.edu.cn&ifnotice=1&rindex=5)). If published, this will include your full peer review and any attached files.

If you choose “no”, your identity will remain anonymous but your review may still be made public.

**Do you want your identity to be public for this peer review?** For information about this choice, including consent withdrawal, please see our [Privacy Policy](https://mailgw.whu.edu.cn/linkserver?dest=aHR0cHM6Ly90cmFjay5lZGl0b3JpYWxtYW5hZ2VyLmNvbS9DTDAvaHR0cHM6JTJGJTJGd3d3LnBs%0D%0Ab3Mub3JnJTJGcHJpdmFjeS1wb2xpY3kvMS8wMTBmMDE5ZjY4N2Q1NTY1LTQ3NmRlNjFkLWJjZjkt%0D%0ANDY4OC04NGVkLTU4NjFmYjdhMDk0YS0wMDAwMDAveVhvblhDSEs5SzUyX2VOdFI2WGM2ZkdKdnFM%0D%0ALWVtblBVcmRYN2t2aTgzVT0yNTg=%0D%0A&tid=_____wC3Jth8MFhq645mAA--.7S2&rcpt=yuxi2018@whu.edu.cn&ifnotice=1&rindex=6).

Reviewer #1: No

Reviewer #2: No

[NOTE: If reviewer comments were submitted as an attachment file, they will be attached to this email and accessible via the submission site. Please log into your account, locate the manuscript record, and check for the action link "View Attachments". If this link does not appear, there are no attachment files.]

To ensure your figures meet our technical requirements, please review our figure guidelines: https://journals.plos.org/plosone/s/figures

You may also use PLOS’s free figure tool, NAAS, to help you prepare publication quality figures: https://journals.plos.org/plosone/s/figures#loc-tools-for-figure-preparation.

NAAS will assess whether your figures meet our technical requirements by comparing each figure against our figure specifications.
